# Sustainable Green Synthesis of Fe_3_O_4_ Nanocatalysts for Efficient Oxygen Evolution Reaction

**DOI:** 10.3390/nano15171317

**Published:** 2025-08-27

**Authors:** Erico R. Carmona, Anandhakumar Sukeri, Ronald Nelson, Cynthia Rojo, Arnoldo Vizcarra, Aliro Villacorta, Felipe Carevic, Ricard Marcos, Bernardo Arriaza, Nelson Lara, Tamara Martinez, Lucas Patricio Hernández-Saravia

**Affiliations:** 1 Laboratorio de Bionanomateriales, Facultad de Recursos Naturales Renovables, Universidad Arturo Prat, Campus Huayquique, Iquique 1100000, Chilefcarevic@unap.cl (F.C.); 2 Núcleo de Investigación Aplicada e Innovación en Ciencias Biológicas, Facultad de Recursos Naturales Renovables, Universidad Arturo Prat, Av. Arturo Prat s/n, Campus Huayquique, Iquique 1100000, Chile; 3 Department of Chemistry, Faculty of Engineering and Technology, SRM Institute of Science and Technology, Kattankulathur 630 203, Tamil Nadu, India; 4 Departamento de Química, Facultad de Ciencias, Universidad Católica del Norte, Avda. Angamos 0610, Antofagasta 1270709, Chile; rnelson@ucn.cl (R.N.);; 5 Campus Velasquez, Universidad de Tarapacá, Arica 1000007, Chile; 6 Grup de Mutagenesi, Departament de Genética i de Microbiologia, Facultat de Biociencies, Universitat Autònoma de Barcelona, 08193 Barcelona, Spain; ricard.marcos@uab.cat

**Keywords:** green synthesis, Fe_3_O_4_ nanoparticles, Electrocatalysis, oxygen evolution reaction, Sustainability

## Abstract

This work focuses on the sustainable green synthesis of magnetic iron oxide nanoparticles (Fe_3_O_4_NPs) using bioreductants derived from orange peel extracts for application in the efficient oxygen evolution reactions (OER). The synthesized catalysts were characterized using X-ray diffraction analysis, field emission scanning electron microscopy (FESEM), energy dispersive X-ray analysis (EDS), transmission electron microscopy (TEM), Fourier transform infrared spectroscopy (FTIR), and UV–visible spectroscopy. The Fe_3_O_4_NPs exhibit a well-defined spherical morphology with a larger Brunauer–Emmett–Teller surface area and a significant electrochemically active surface area. The green synthesis using orange peel extracts leads to an excellent electrocatalytic activity of the apparent spherical Fe_3_O_4_NPs (diameter of 9.62 ± 0.07 nm), which is explored for OER in an alkaline medium (1.0 M KOH) using linear-sweep and cyclic voltammetry techniques. These nanoparticles achieved a benchmark current density of 10 mA cm^−2^ at a low overpotential of 0.3 V versus RHE, along with notable durability and stability. The outstanding OER electrocatalytic activity is attributed to their unique morphology, which offers large surface area and an ideal porous structure that enhances the adsorption and activation of reactive species. Furthermore, structural defects within the nanoparticles facilitate efficient electron transfer and migration of these species, further accelerating the OER process.

## 1. Introduction

In recent years, the world’s population has increased exponentially, resulting in a significant rise in CO_2_ emissions from fossil fuels [1]. This has had a significant impact on the environment, which is why there is global interest in developing renewable and sustainable fuels that emit minimal to zero greenhouse gases [2]. The electrochemical splitting [3] of water is one of the most attractive methods for hydrogen and oxygen generation with zero-carbon emission. In this process, the hydrogen evolution reaction (HER) and oxygen reduction reaction (ORR) [4] occur in the cathode [5,6,7,8], while the oxygen evolution reaction (OER) takes place in the anode. However, the sluggish OER kinetics at the anode affect the overall potential of the water splitting. In other words, a high potential must be applied for the transfer of the four electrons of the reaction, as described by the half-reaction 2H_2_O → O_2_ + 4H^+^ + 4e^−^ (E° = 1.23 V at pH = 0), making this process highly inefficient [9]. Therefore, the development of new materials with high electrocatalytic efficiency and stability is essential for CO_2_-free sustainable fuel production.

The current anode used in OER relies on electrocatalysts made from noble metals such as Pt, or noble metal oxides such as RuO_2_ and IrO_2_ [10,11,12]. These materials are not only scarce and expensive, but their electrocatalytic performance is often limited to high current densities. As a result, scaling up this process for industrial applications is not feasible [13,14]. To overcome these challenges, it is crucial to develop highly stable, efficient, cost-effective catalysts that possess superior catalytic activity [15]. Recently, several groups have investigated different nanomaterials for OER [16]; however, transition metals are a popular choice due to their low cost and abundance, such as Co_3_O_4_NPs [17], CoEDA [18], Mn_3_O_4_ [19], Ni/NiSPx/NF [20], WCx/CC [21], NiFe-LDH [22], Ni-MOF/NiFe_2_O_4_ [23], 3NiFe-1N-GA-800 [24], and FeCoNiCuMo-O [25], among others. Among these, magnetite nanoparticles (Fe_3_O_4_ NPs) have garnered significant interest for OER [26,27,28,29,30] due to their excellent magnetic and catalytic properties, as well as their low cost and abundance [31,32].

Furthermore, it is essential to evaluate the different synthesis methods for preparing Fe_3_O_4_ NPs. Several approaches are available, including solvothermal [33,34], ultrasonic/co-precipitation [35], co-precipitation [36], hydrothermal [37], sol–gel [38], microemulsion [39], and electrospinning [40]. However, despite the wide range of available methods for synthesizing Fe_3_O_4_NPs, many of them involve high costs, complex procedures, and the generation of toxic waste. In contrast, sustainable and ecological methods offer an attractive alternative, reducing environmental impact and promoting more environmentally friendly practices [41,42]. Nanomaterials with diverse sizes and shapes have been prepared through green synthesis [43,44], using different plant-based extracts as bioreductants. These include organic waste [45], *P. benghalensis* leaves [46], banana peels (*Musa paradisiaca*) [47], black tea leaves (*Camellia sinensis*) [48], *Citrus medica* Linn. (Idilimbu) juice [49], lemon peels [50], mango extract [51], and orange peels [52]. Also, it is worth noting that the global production of oranges is approximately 52 million tons, which results in a significant amount of organic waste in the form of orange peels. Additionally, iron is abundantly present in oranges. In this study, we propose an environmentally friendly, simple, and sustainable approach for producing Fe_3_O_4_ NPs using orange peel residues as bio-reducing agents for efficient OER.

## 2. Results

### 2.1. Materials Characterization Using X-Ray Diffraction and Microscopy Analysis

**Figure 1:**
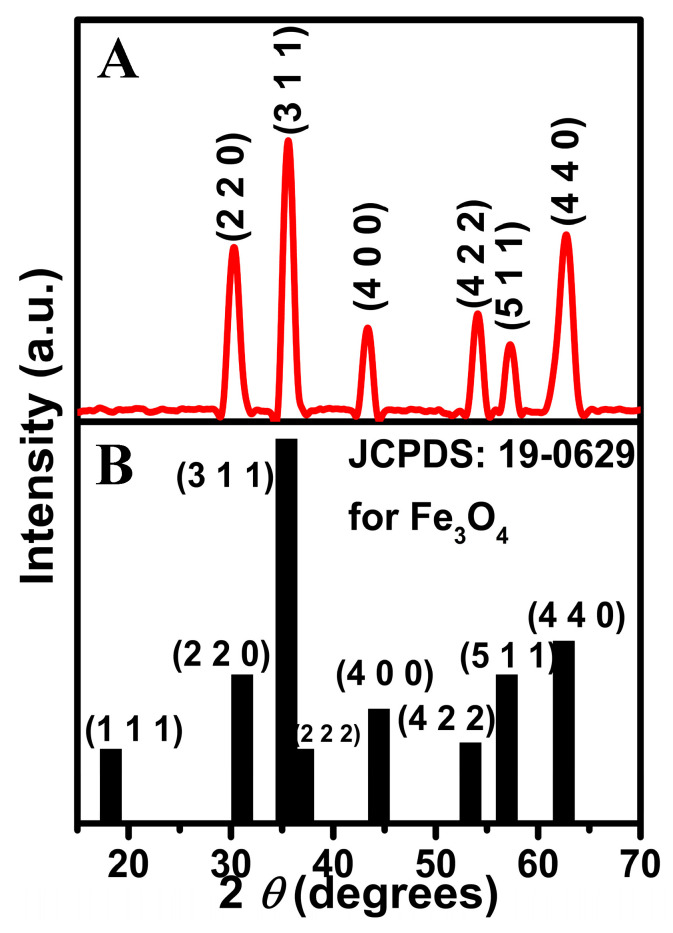
(**A**) XRD pattern of the synthesized Fe_3_O_4_ NPs; (**B**) corresponding JCPDS file: 19-0629.

Figure 1 shows the XRD pattern of magnetite Fe_3_O_4_. The obtained 2θvalues are 30.2° (200), 35.6° (311), 43.4° (400), 57.3° (511), and 62.8° (440), in agreement with previous reports, confirming the cubic spinel structure of magnetite Fe_3_O_4_. These peaks are consistent with studies carried out by Selvaraj et al. [53] and Elizondo et al. [54], indicating that the peaks at 2θ values of 43.4° and 57.3° correspond to (400) and (511) crystalline planes of Fe_3_O_4_ with rhombohedral structures. In addition, such results are also consistent with the reference JCPDS (19-0629) pattern of magnetite Fe_3_O_4_ (see Figure 1A,B).

**Figure 2:**
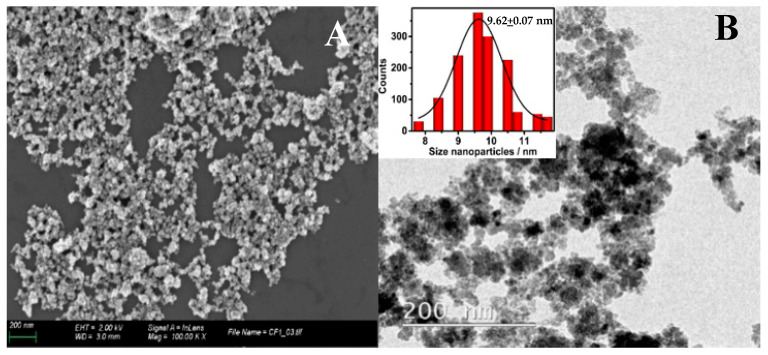
(**A**) SEM and (**B**) TEM image of Fe_3_O_4_ NPs (inset: size distribution histogram).

The surface morphology of the Fe_3_O_4_ NPs was analyzed by SEM and TEM analysis (Figure 2A,B). The images reveal a predominantly spherical morphology, and the particle size distribution histogram (Inset, Figure 2B) shows particle sizes between 7.8 nm and 11.7 nm, with an average size of 9.62 ± 0.07 nm. The nanoparticle size obtained in this study is smaller than those reported for Fe_3_O_4_ NPs synthesized by other methods (Table S1).

**Figure 3:**
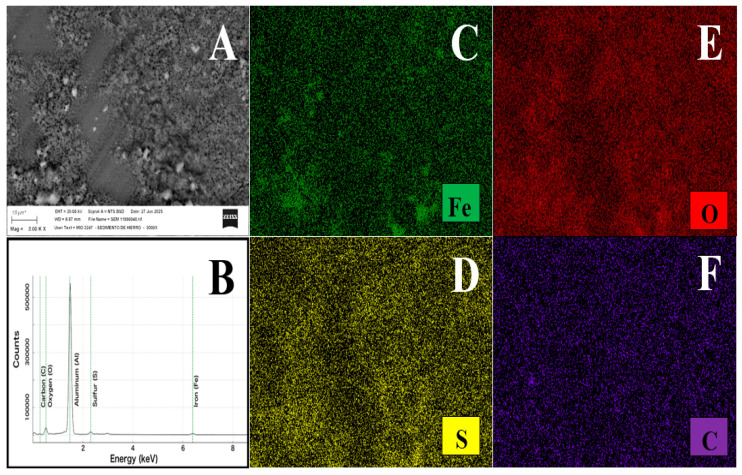
The SEM image of Fe_3_O_4_ NPs (**A**), EDX analysis and its elemental mapping for Fe_3_O_4_ NPs (**B**), and the presence of Fe (**C**), S (**D**), O (**E**), and C (**F**) atoms in the Fe_3_O_4_ NPs.

Additionally, the elemental composition of the Fe_3_O_4_ NPs was analyzed using energy-dispersive X-ray (EDX) analysis along with elemental mapping (Figure 3). The results show strong signals in the oxygen and iron regions, confirming the presence of Fe_3_O_4_ NPs. Figure S1 corroborated the presence of Fe and O, indicating the expected chemical composition of the Fe_3_O_4_NPs; a signal for aluminum was also detected, attributed to the sample holder. Moreover, signals corresponding to carbon (C), oxygen (O), and sulfur (S) were observed, which is likely due to the presence of polyphenols from the organic extracts capping the Fe_3_O_4_ NPs, thereby contributing to their stabilization [55].

### 2.2. UV–Visible and FT-IR Analysis

**Figure 4:**
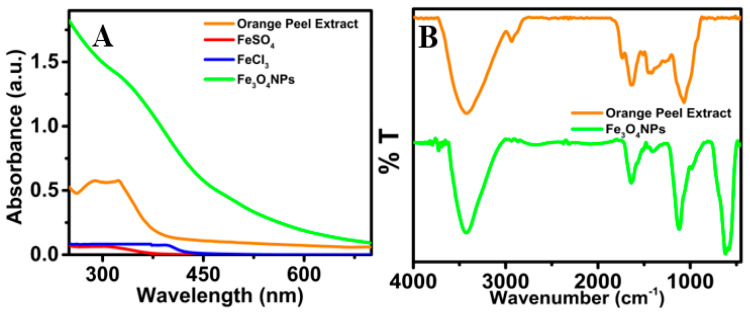
(**A**) UV–vis absorption spectra of Fe_3_O_4_ NPs synthesized with 0.5% of orange peel extract and Fe salts (**B**); FTIR spectra of Fe_3_O_4_ NPs synthesized using orange peel extract (green line) and orange peel extract (orange line).

The results of the UV–visible characterization of the synthesized Fe_3_O_4_ NPs are shown in Figure 4A. Absorption peaks observed at 240, 270, and 340 nm indicate the presence of polyphenols from the orange peel extract. These results are consistent with previous reports by Zayed et al. [56], who observed similar peaks related to the polyphenol content in plant leaf infusions. In comparison with the Fe^2+^/Fe^3+^ precursor salts, the absorbance peak at 350 nm (green line in Figure 4A) suggests the reduction in Fe ions and supports the formation of Fe_3_O_4_ NPs [52,57,58].

Figure 4B presents the FTIR spectra of Fe_3_O_4_ NPs and the orange peel extract. The main absorption bands observed at 547, 1033, 1635, and 3540 cm^−1^ (see Table 1) are attributed to the Fe-O, S=O, C=C, and –OH, respectively [59,60]. Notably, the Fe-O characteristic peak (547 cm^−1^) appeared only in the Fe_3_O_4_ NPs spectrum.

### 2.3. Prepared Fe_3_O_4_NPs by BET Analysis

Table 2 shows the measured specific surface area (SSA) of the Fe_3_O_4_ nanoparticles (NPs), which was found to be 80.37 m^2^ g^−1^. The total pore volume (PV) of Fe_3_O_4_NPs was 0.274 cm^3^ g^−1^, and the average PS value recorded was 4.66 nm. The high pore volume indicates a high degree of porosity, suggesting a large number of sites, which favors the electrocatalytic performance of the Fe_3_O_4_NPs. The BET surface area of Fe_3_O_4_NPs was calculated to be 80.37 m^2^ g^−1^, considerably higher than the values reported by various chemical and green synthesis methods (Table S2).

### 2.4. Electrochemical Characterization of Fe_3_O_4_NPs

The larger accessible surface area of Fe_3_O_4_NPs indicates their superior electron-transferring capabilities during redox reactions. To further investigate this, the electrochemical active surface area (ECSA) of the Fe_3_O_4_NPs was calculated using the Randles–Sevcik Equation (1) [61].

(1) $$i_{p}=(2.69\times 10^{5})n^{3/2}D^{1/2}\nu^{1/2}AC$$

**Figure 5:**
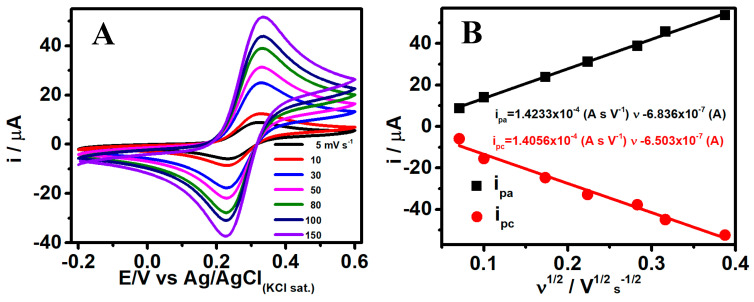
(**A**) CVs of Fe_3_O_4_NPs at different scan rates (0.005–0.150 V s^−1^) and (**B**) corresponding linear plot of the square root of scan rate vs. i. The [Fe(CN)_6_]^3−/4−^ system was used for electrochemical characterization studies.

This equation relates the peak current (Ipa) to the number of electrons transferred (*n* = 1), the diffusion coefficient (D) of the [Fe(CN)_6_]^3−/4−^ redox probe (7.60 × 10^−6^ cm^2^ s^−1^), the scan rate (ν), the active surface area (A), and the concentration (C of 1 × 10^−6^ mol cm^−3^). The ECSA of Fe_3_O_4_NPs was determined by measuring the redox peak current across scan rates ranging from 0.005 to 0.150 V s^−1^ (as shown in Figure 5). An increase in the scan rate led to a more pronounced redox peak current response with changes in redox peak potential, which is attributed to rapid electron transport facilitated by the Fe_3_O_4_NPs. Furthermore, a strong linear relationship was observed between the redox peak current and the square root of the scan rate (with correlation coefficients of 0.9988 for I_pa_ and 0.9971 for I_pc_), consistent with a diffusion-controlled electrode process.

From the slope of this linear plot, the ECSA of the Fe_3_O_4_NPs was calculated to be approximately 0.198 cm^2^. This value represents a significant enhancement, being 2.7 times greater than the ECSA of a bare glassy carbon electrode (GCE), which was measured at 0.07065 cm^2^. These findings conclusively demonstrate that Fe_3_O_4_NPs possess both a high surface area and efficient electron transfer properties, suggesting their promising application as an effective electrode modification in electrochemical detection research.

### 2.5. Electrochemical Studies for the Oxygen Evolution Reaction

**Figure 6:**
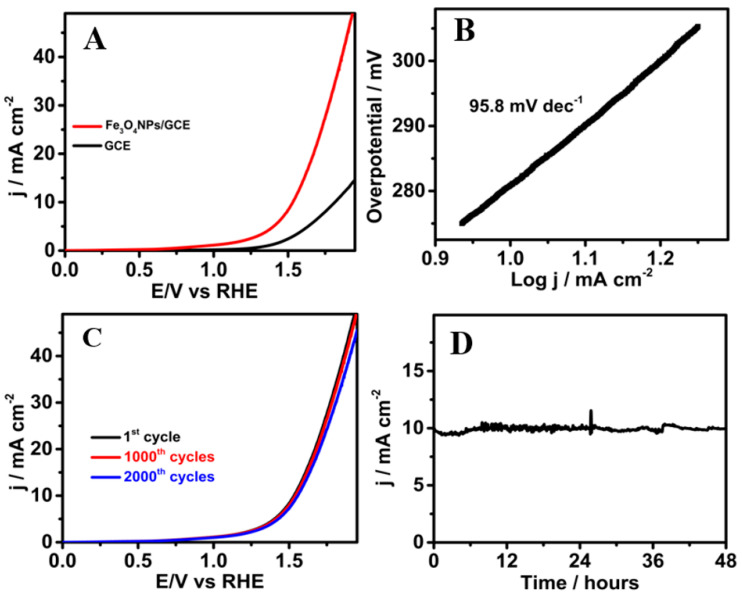
(**A**) LSV polarization curves recorded in 1.0 M KOH solution with GCE (black) and Fe_3_O_4_ NPs/GCE (red) electrodes at the scan rate of 2 mV s^−1^. (**B**) Tafel plot, (**C**) durability in 1.0 M KOH solution after 1000 and 2000 cycles, and (**D**) amperometric curves at an applied overpotential of 0.3 V for Fe_3_O_4_ NPs/GCE.

The electrochemical performance of GCE and Fe_3_O_4_NPs/GCE was evaluated towards oxygen evolution reaction (OER) by recording the linear sweep voltammogram (LSV) in a 1.0 M KOH solution (pH = 14) at the scan rate of 2 mV s^−1^ (Figure 6A). The Fe_3_O_4_ NPs/GCE exhibited significantly enhanced electrocatalytic activity compared to the bare GCE, as evidenced by a lower onset potential and a marked increase in current density. Moreover, the benchmark current density of 10 mA cm^−2^ was achieved at a very low overpotential of 0.3 V vs. RHE, which is 0.28 V less overpotential than GCE (0.58 V). This improved performance can be attributed to the increased surface area and roughness of the Fe_3_O_4_ NPs/GCE, resulting from the small particle size, enlarging the number of active sites and enhancing the electrocatalytic activity of OER. In addition, the Tafel slope was calculated from the Tafel plot (η vs. Log j) using the LSV data obtained from Figure 6B, and the value was found to be 95.8 mV dec^−1^ for Fe_3_O_4_ NPs/GCE. Furthermore, the OER performance of the nanocatalyst was compared with other Fe-based catalysts reported in the literature (Table 3), showing that the present system exhibits notably improved performance relative to previously reported values. The stability of OER at the benchmark current density of 10 mA cm^−2^ was also tested using LSV polarization curves (Figure 6C) and amperometry (Figure 6D). LSV recorded at the 1st, 1000th, and 2000th cycle showed negligible variation in performance, indicating stable electrocatalytic activity over prolonged cycling. In addition, there is no significant change in the current response of 10 mA cm^−2^ over 48 h in the amperometry measurement (at an applied overpotential of 0.3 V). These results demonstrate the efficient durability and stability of Fe_3_O_4_NPs/GCE catalyst for OER. The OER mechanism is complex and can proceed via different pathways, but a common one is the oxide path, which based on the results obtained from Fe_3_O_4_NPs and what was reported by Soni A. et al. [62] suggests that the OER follows the following mechanism:

$$M+{OH}^{-}\to M-OH+\bar{e}$$

$$M-OH+{OH}^{-}\to M-O+H_{2}O+\bar{e}$$

$$M-O\to 2M+O_{2}$$

## 3. Materials and Methods

### 3.1. Chemicals

Iron (II) sulfate (FeSO_4_), iron (III) chloride (FeCl_3_), Nafion (5 wt%), ethanol (C_2_H_5_OH), and potassium hydroxide (KOH) were used as received from Sigma-Aldrich and Merck, respectively. High-purity argon and oxygen gases were purchased from local suppliers. Unless otherwise stated, all the aqueous solutions were prepared using Milli-Q water with 18.0 MΩ cm resistivity.

### 3.2. Instruments

Electrochemical measurements were carried out using an Autolab Origaflex OGF 01A potentiostat with OrigaMaster 5 software. A glassy carbon electrode (GCE) and/or a Fe_3_O_4_ nanoparticles-modified glassy carbon electrode (Fe_3_O_4_ NPs/GCE) was used as working electrode, while an Ag/AgCl electrode (saturated KCl) and a platinum wire served as the reference and counter electrodes, respectively. All potentials in this work are reported vs. the reversible hydrogen electrode (RHE), where E (vs. RHE) = E (vs. Ag/AgCl) + 0.0592 × pH + 0.197 V at 25 °C. The formation of Fe_3_O_4_ nanoparticles was monitored with a UV–visible spectrophotometer (Spectroquant^®^ Prove 300, Merck, Germany), operating with a 1 nm interval and integration time of 1 s, over a wavelength a range of 200–800 nm. FTIR analyses were conducted using an FT/IR 4X spectrometer (Jasco, USA). The samples were analyzed using an ATR accessory to examine the surface functional groups of the reduced Fe_3_O_4_NPs. The FTIR spectra were collected at a spatial resolution of 4 cm^−1^ in the transmission mode, between 4000 and 400 cm^−1^. The XRD analysis of the samples was carried out in an X-ray diffractometer (Bruker, Model D8 Advance, USA) using a Vertical Bragg–Brentano goniometer, a solid-state detector (Centelleo Model), with Cu Ka radiation operating at 40 kV and 30 mA. The topography of the synthesized Fe_3_O_4_ NPs was examined using a JEM 1400 transmission electron microscope (JEOL Ltd., Tokyo, Japan). For TEM analysis, Fe_3_O_4_ NPs were re-suspended in water at a concentration of 0.1 mg mL^−1^, sonicated for 30 min, and 100 μL of the suspension was mixed with silver epoxy adhesive and deposited on a silica film. The sample was then dried in an oven at 60 °C until complete evaporation of water, prior to TEM imaging. Surface characterization of Fe_3_O_4_ NPs was conducted using an SEM instrument (Zeiss Merlin, Oberkochen, Germany). The processing of SEM micrographs was interpreted using ImageJ win32 software.

### 3.3. Preparation of Orange Peel Extract

The orange peels were thoroughly washed with deionized water to remove any surface impurities. They were then dried in an oven at 60 °C for 36 h. Once dried, the peels were crushed with a mini blender until a fine powder of 5 µm ± 0.5 µm was obtained. Subsequently, the orange peel extract was prepared by dispersing 40 g of powdered peel per liter of deionized water, followed by stirring at 1000 rpm at 80 °C for 2 h. The resulting extract was then filtered and stored at −4 °C.

### 3.4. Green Synthesis of Fe_3_O_4_ Nanoparticles

**Scheme 1:**
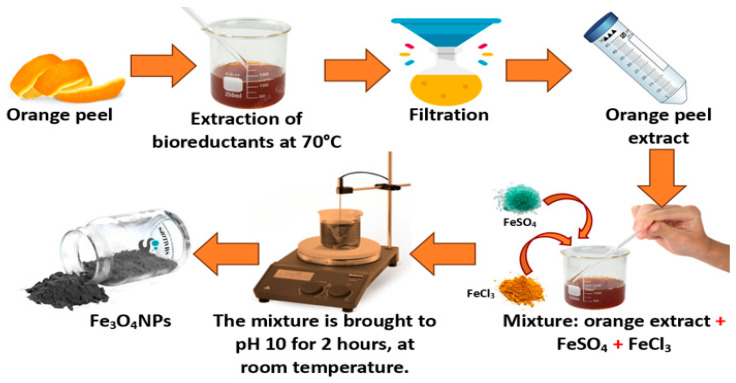
Fabrication procedure of Fe_3_O_4_ NPs using orange peel extracts by eco-friendly green synthesis method.

The green synthesis of Fe_3_O_4_ NPs was carried out by mixing FeSO_4_ and FeCl_3_ salts in a 2:1 ratio of 10 mM. Under constant stirring, the orange peel extract was added dropwise to the Fe salts solution, and the pH was adjusted to 10. The solution was stirred for 30 min at 300 rpm. Once this process was finished, the final solution was allowed to decant to remove the supernatant. The resulting Fe_3_O_4_ NPs were collected, washed with ethanol, and dried in an oven at 60 °C for 24 h, as shown in Scheme 1.

### 3.5. Preparation of Fe_3_O_4_ NPs Modified Glassy Carbon Electrode (GCE)

The GCE was carefully polished using a polishing cloth and 0.05 µm alumina powder, followed by sonication for 15 min in an aqueous solution. Fe_3_O_4_ NPs (2 mg mL^−1^) were dispersed in water, and Nafion (0.1%) solution was added. The mixture was sonicated for 10 min to obtain a homogeneous suspension. Subsequently, 10 μL of the suspension was drop-cast on the surface of the GCE and allowed to dry at room temperature.

## 4. Conclusions

This work successfully demonstrates the sustainable green synthesis of Fe_3_O_4_ nanocatalysts using orange peel extracts, which exhibit excellent electrocatalytic activity for the OER. These nanoparticles, with an average diameter of 9.62 ± 0.07 nm, achieve a benchmark current density of 10 mA cm^−2^ at a low overpotential of 0.3 V versus RHE, along with remarkable durability and stability. This high performance is attributed to their unique morphology, large surface area, and porous structure, which facilitate efficient adsorption, activation of reactive species, and electron transfer. These findings present a promising pathway for developing novel, cost-effective, and efficient Fe-based nanocatalysts for applications in energy conversion, sustainable chemistry, and electrochemistry.

## Figures and Tables

**Table 1:** FTIR analysis of Fe_3_O_4_NPs and orange peel extract containing possible functional groups.

Fe_3_O_4_ NPs Wavenumber (cm^−1^)	Functional Groups	Orange Peel Extract Wavenumber (cm^−1^)	Functional Groups
547	Strong Fe–O	547	-
1033	Medium S=O	1033	Medium S=O
1635	Medium C=C	1635	Medium C=C
3540	Board O–H	3540	Board O–H

**Table 2:** The tabulated values of BET surface area (m^2^ g^−1^), pore volume (cm^3^ g^−1^), and pore size (nm) of Fe_3_O_4_NPs.

Nanoparticles	BET Surface Area (m^2^ g^−1^)	Pore Volume (cm^3^ g^−1^)	Pore Size (nm)
Fe_3_O_4_NPs	80.37	0.274	4.66

**Table 3:** Comparison of Fe-based nanocatalysts for OER performances.

Materials	Electrolytes	Overpotential (V) (at 10 mA cm^−2^)	Ref.
(NiFe)S_2_	1.0 M KOH	0.32	[63]
porous Ni–FeO	1.0 M KOH	0.33	[64]
NiFe@NC	1.0 M KOH	0.58	[65]
FePO_4_/NF	1.0 M KOH	0.31	[66]
Fe-doped NiO_x_	1.0 M KOH	0.31	[67]
Ni_0.62_Fe_0.38_P	1.0 M KOH	0.31	[68]
Ni_1.5_Fe_0.5_P/CF	1.0 M KOH	0.36	[69]
S-NiFe_2_O_4_/NF	1.0 M KOH	0.42	[70]
NiFeOCPc@rGO-K	1.0 M KOH	0.37	[9]
Fe_0.22_Ni_0.78_(OH)_2_	1.0 M KOH	0.32	[71]
NiO_x_–NiO_x_/FeO_x_	1.0 M KOH	0.32	[72]
Fe_3_O_4_/graphene	0.1 M KOH	-	[73]
FeCoV-NiMOF	0.1 M KOH	0.238	[74]
Fe_3_O_4_-600	1.0 M NaOH	0.33	[28]
**Fe_3_O_4_NPs**	**1.0 M KOH**	**0.30**	**This work**

## Data Availability

Data are contained within the article and Supplementary Materials.

## Supplementary Materials

The following supporting information can be downloaded at https://www.mdpi.com/article/10.3390/nano15171317/s1, Table S1. Size and shape comparison of the synthesized Fe_3_O_4_ nanocatalyst with the literature. Figure S1. SEM image of elemental mapping distribution Fe_3_O_4_NPs for Fe, O, S and C respectively.
