# Supplementary material for: Sustainable Green Synthesis of Fe3O4 Nanocatalysts for Efficient Oxygen Evolution Reaction
Source: Nanomaterials (Basel). 2025 Aug 27;15(17):1317. doi: 10.3390/nano15171317 (PMC12430110; doi:10.3390/nano15171317)
Supplement: Supplementary file 1 [file nanomaterials-15-01317-s001.zip › nanomaterials-3737491-supplementary.pdf]

Sustainable green synthesis of Fe_3O_4 nanocatalyst for efficient oxygen evolution reaction

Erico R. Carmona [1,2], Anandhakumar Sukeri [3,*], Ronald Nelson [4], Cynthia Rojo [1,2], Arnoldo Vizcarra [5], Aliro Villacorta [1,2], Felipe Carevic [1,2], Ricard Marcos [6], Bernardo Arriaza [5], Nelson Lara [5], Tamara Martinez [4] and Lucas Patricio Hernández-Saravia [1,2,*]

1. Laboratorio de Bionanomateriales, Facultad de Recursos Naturales Renovables, Universidad Arturo Prat, Campus Huayquique, Iquique 1100000, Chile; fcarevic@unap.cl (F.C.)
2. Núcleo de Investigación Aplicada e Innovación en Ciencias Biológicas, Facultad de Recursos Naturales Re-novables, Universidad Arturo Prat, Av. Arturo Prat s/n, Campus Huayquique, Iquique 1100000, Chile
3. Department of Chemistry, Faculty of Engineering and Technology, SRM Institute of Science and Technology, Kattankulathur 630 203, Tamil Nadu, India
4. Departamento de Química, Facultad de Ciencias, Universidad Católica del Norte, Avda. Angamos 0610, Antofagasta 1270709, Chile; rnelson@ucn.cl (R.N.)
5. Universidad de Tarapacá, Arica 1000007; nlara@academicos.uta.cl (N.L.)
6. Grup de Mutagenesi, Departament de Genética i de Microbiologia, Facultat de Biociencies, Universitat Autònoma de Barcelona, 08193 Barcelona, Spain; ricard.marcos@uab.cat

Correspondence: anandhas2@srmist.edu.in (A.S.); luhernande@unap.cl (L.P.H.-S.)

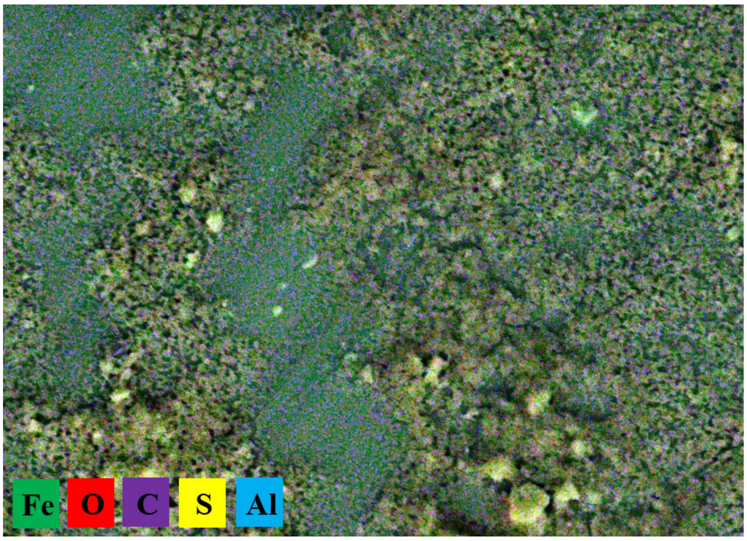

Figure S1. SEM image of elemental mapping distribution Fe_3O_4NPs for Fe, O, S and C respectively.

Table S1. Size and shape comparison of the synthesized Fe_3O_4 nanocatalyst with the literature.

Synthesis method	Nanoparticles	Size (nm)	Morphology	Reference
Precipitation method	Fe_3O_4NPs	40	Sphere	[1]
Sonochemical method	Fe_3O_4NPs	60	Sphere	[2]
Electrochemical and hydrothermal method	Fe_3O_4NPs	~50	Sphere	[3]
Hydrothermal synthesis	Fe_3O_4NPs	24.88	Sphere	[4]
Polyol method	Fe_3O_4NPs	13.72	Sphere	[5]
Solvothermal synthesis	Fe_3O_4NPs	18.5	Sphere	[6]
Green hydrothermal synthesis	Fe_3O_4NPs	74.43	Sphere	[7]
Green synthesis (*Citrus aurantium*)	Fe_3O_4NPs	12.5	Sphere	[8]
Green synthesis (*Garcinia mangostana*)	Fe_3O_4NPs	13.42 ± 1.58	Sphere	[9]
Green synthesis (*Allium sativum*)	Fe_3O_4NPs	70.223	Sphere	[10]
Sustainable green synthesis (***orange peel extract***) **approach**	**Fe_3O_4 NPs**	**9.62 ± 0.07**	**Sphere**	**This work**

References

1. Nabiyouni, G.; Julaee, M.; Ghanbari, D.; Aliabadi, P.C.; Safaie, N. Room Temperature Synthesis and Magnetic Property Studies of Fe_3O_4 Nanoparticles Prepared by a Simple Precipitation Method. *J. Ind. Eng. Chem.* **2015**, *21*, 599–603, doi:10.1016/j.jiec.2014.03.025.
2. Ghanbari, D.; Salavati-Niasari, M.; Ghasemi-Kooch, M. A Sonochemical Method for Synthesis of Fe_3O_4 Nanoparticles and Thermal Stable PVA-Based Magnetic Nanocomposite. *J. Ind. Eng. Chem.* **2014**, *20*, 3970–3974, doi:10.1016/j.jiec.2013.12.098.
3. Kalidass, J.; Reji, M.; Sivasankar, T. Synthesis of Fe_3O_4 Nanoparticles with Enhanced Properties via Sonoelectrochemical Approach: A Comparative Study with Electrochemical and Hydrothermal Method. *Chem. Eng. Process. - Process Intensif.* **2024**, *197*, 109690, doi:10.1016/j.cep.2024.109690.
4. Rafie, S.F.; Sayahi, H.; Abdollahi, H.; Abu-Zahra, N. Hydrothermal Synthesis of Fe_3O_4 Nanoparticles at Different PHs and Its Effect on Discoloration of Methylene Blue: Evaluation of Alternatives by TOPSIS Method. *Mater. Today Commun.* **2023**, *37*, 107589, doi:10.1016/j.mtcomm.2023.107589.
5. Oh, A.H.; Park, H.-Y.; Jung, Y.-G.; Choi, S.-C.; An, G.S. Synthesis of Fe_3O_4 Nanoparticles of Various Size via the Polyol Method. *Ceram. Int.* **2020**, *46*, 10723–10728, doi:10.1016/j.ceramint.2020.01.080.
6. Fotukian, S.M.; Barati, A.; Soleymani, M.; Alizadeh, A.M. Solvothermal Synthesis of

$CuFe_2O_4$ and Fe_3O_4 Nanoparticles with High Heating Efficiency for Magnetic Hyperthermia Application. *J. Alloys Compd.* **2020**, *816*, 152548, doi:10.1016/j.jallcom.2019.152548.

7. Yaghoobi, M.; Asjadi, F.; Sanikhani, M. A Facile One-Step Green Hydrothermal Synthesis of Paramagnetic Fe_3O_4 Nanoparticles with Highly Efficient Dye Removal. *J. Taiwan Inst. Chem. Eng.* **2023**, *144*, 104774, doi:10.1016/j.jtice.2023.104774.
8. Bassim, S.; Mageed, A.K.; AbdulRazak, A.A.; Majdi, H.S. Green Synthesis of Fe_3O_4 Nanoparticles and Its Applications in Wastewater Treatment. *Inorganics* **2022**, *10*, 260, doi:10.3390/inorganics10120260.
9. Yusefi, M.; Shameli, K.; Su Yee, O.; Teow, S.-Y.; Hedayatnasab, Z.; Jahangirian, H.; Webster, T.J.; Kuča, K. Green Synthesis of Fe_3O_4 Nanoparticles Stabilized by a Garcinia Mangostana Fruit Peel Extract for Hyperthermia and Anticancer Activities. *Int. J. Nanomedicine* **2021**, *Volume 16*, 2515–2532, doi:10.2147/IJN.S284134.
10. Liu, L.; Li, Y.; AL-Huqail, A.A.; Ali, E.; Alkhalifah, T.; Alturise, F.; Ali, H.E. Green Synthesis of Fe_3O_4 Nanoparticles Using Alliaceae Waste (Allium Sativum) for a Sustainable Landscape Enhancement Using Support Vector Regression. *Chemosphere* **2023**, *334*, 138638, doi:10.1016/j.chemosphere.2023.138638.
